# Bio-Based and Biodegradable Plastics: From Passive Barrier to Active Packaging Behavior

**DOI:** 10.3390/polym12071537

**Published:** 2020-07-12

**Authors:** Alexey Iordanskii

**Affiliations:** Semenov Institute of Chemical Physics, Russian Academy of Sciences, 119991 Moscow, Russia; aljordan08@gmail.com

**Keywords:** bio-based polymers, biodegradable packaging, diffusion, permeability, mechanical behavior, biopolymer structure, encapsulation, life cycle analysis

## Abstract

An overview of the articles has presented for the Special Issue “Bio-Based and Biodegradable Plastics: From Passive Barrier to Active Packaging Behavior”. This issue has objective of collecting comprehensive findings regarding structure and functionality of bio-based sustainable polymers performing as multifaceted barrier and packaging in food, cosmetic, and other areas. The content of the collection covers diverse fields of knowledge embracing polymer chemistry, materials science, transport–diffusion phenomena, biodegradation exploring, and others.

The unprecedented energy crisis, triggered by the pandemic, in combination with environmental safety deterioration, has stimulated the accelerated transition from the synthetic bio-stable plastics from hydrocarbon fossils to the bio-based and biodegradable plastics produced from natural renewable resources. In line with the European Strategy for Bioplastics in a Circular Economy, the rational design of biopolymers has to provide them with the desired functionality, sustainability, and nonharmful utility. Mostly affected by this revolutionary trend, biodegradable polymers and their composites must be applied in the areas of packaging manufacturing and especially food packaging implementation. In the framework of this Special Issue the invited authors have presented their significant contributions to augmenting the current trends in barrier and packaging materials with the special characteristics of biodegradable plastics.

As bio-based and biodegradable polymers, polyhydroxyalkanoates, with their basic representative, poly (3-hydroxybutyrate) (PHB), and polylactides (PLA) are the main candidates to replace fossil-based polymer materials especially in the packaging industry. The intention of the comprehensive paper by Siracusa et al. [1] was to manufacture, via blending, fully biodegradable barriers against atmospheric gases and to first combine diffusivity data with blend segmental mobility. A multifaceted approach combining dynamic (permeation techniques and probe-radical ESR methodology) and structural (SEM, DSC, and colorimetry) methods is given to describe the complex behavior of fully bio-based blends with different compositions. Both probe rotation mobility, measured by the ESR method, and atmospheric gas transport characteristics monotonically decreased in the PLA/PHB blend barriers because they were transferred from PLA with lower crystallinity to PHB with a higher one. These findings, in combination with the structure-morphology pattern, could be interpreted as ground barrier packaging characteristics, which are necessary for a coherent interpretation of recently elaborated packaging with active functions such as environmental safety, antimicrobial performance, and the controlled release of food-modifiers.

A comprehensive study considering the physicochemical and mechanical behavior of pristine poly (3-hydroxybutyrate) PHB and copolymers of 3-hydroxybutyrate (HB) with 3-hydroxyvalerate (HV) during long-term enzymatic and hydrolytic biodegradation is presented by Bonartsev et al. [2]. The evolution of principal characteristics such as polymer weight, its average molecular weight, crystallinity, and surface hydrophobicity as function of the HB/HV ratio is carefully described. It is interesting that on the scale of monomer content there is a critical point at ~5.7–5.9% of HV content that could be treated as a transition point for sharp changes in the structural and mechanical properties of the copolymer films.

Along with copolymer synthesis of biodegradable barriers, there is an alternative method of novel packaging design that is based on environmentally safe technology, namely solid-state extrusion without the use of toxic solvents. Rogovina et al. [3] present a prominent biodegradable blend on the base of polylactide (PLA) and PHB. The thermoplastic biopolyesters were obtained by blending under shear deformations and electrospinning methods in the form of films and ultrathin electrospun fibers. The crystallinity and the specific thermal transitions (glass transition, cold crystallization, and melting) for the individual PLA, PHB, and their compositions with polyethylene glycol were analyzed. Thermal and mechanical features of the ternary blends testified the pronounced behavior of PEG as the plasticizer with the noticeable increase in tensile characteristics and with a remarkable decrease in glass-transition temperature.

Chalykh et al. [4] address the topic of water vapor permeability through porous polymeric membranes presenting synthetic and natural transport barriers with a diverse hydrophilic–hydrophobic balance. At hydrolysis and enzymatic attack, water molecules promote macromolecular degradation and therefore affect the functional behavior of barrier and packaging materials. Barrier characterization is also critical for understanding the role of water diffusion and sorption in packaging matrices as the control factors that contribute to bacterial growth and food deterioration. In addition, another important point that water sorption capacity reveals is moisture’s impact on gas permeability and selectivity. The prospects of this effect should be thoroughly studied by experts in future publications.

Kildeeva et al. [5] present a circumstantial description of chitosan crosslinked by genipin (Gp) with the aim of depressing polysaccharide solubility and expand the array of functionalities that, along with versatile eco- and bio-compatibility, open up promising prospects in the packaging industry. The Gp as the natural bifunctional reagent improves water resistance and mechanical behavior. Water diffusivity, sorption capacity, and the modeling of isotherms, namely their decomposition into the Langmuir and Flory–Huggins constituents, have shown the complicated pattern of water–crosslinked chitosan interaction. The results of this contribution could be useful for food packaging, edible coatings, or for medical applications to improve tensile parameters and to control moisture absorption.

By an international Spain-UK team, in the manuscript of Quiles-Carrillo et al. [6], the formulation of bio-based high-density polyethylene (BHDPE) with gallic acid (GA) was first melt-compounded and comprehensively investigated. The GA-loaded bio-HDPE films produced by extrusion and cast rolling successively were characterized in terms of their mechanical, morphological, and thermal performance as well as ultraviolet (UV) light stability to evaluate their potential application in food packaging. The encapsulation of the natural antioxidant has essentially improved prospects for the thermal and UV light stability of green BHDPE and, very probably, of other green polyolefins.

Kirsh et al. [7] carefully analyzed the binary (polyethylene (PE) and birch bark extract (BBE)) and ternary (PE, modified starch, and BBE) blends that are designated for novel food packaging. The innovative idea of bend packaging preparation includes the combination of extrusion and ultrasound treating simultaneously. This approach allowed the authors to obtain barrier films with a uniform distribution of components and increased the fluidity of composite blends as well. Embedding the BBE modifies water vapor and oxygen permeabilities. Besides enhancing the antibacterial activity, the natural extract affects the term of biodegradability and simultaneously improves the tensile and elongation characteristics of barrier films.

An ingenious approach to novel composites for food and agriculture packaging is proposed by Mastalygina et al. [8]. In contrast to the mentioned above work of Quiles-Carrillo where composites on the base of bio-HDPE were considered, here, the authors used a kind of “hybrid” material combining synthetic low density PE and natural rubber (NR). A mycological test with fungi and a full-scale soil test clearly showed that 30 wt% of NR affected PE biodegradability via enhancing its hydrophilicity that was affirmed by a water absorption technique. Additional testing by IR spectroscopy and the DSC technique showed the structural evolution of LDPE after exposure to soil and corresponding weight loss by 7.2% for three months of testing. In addition, the composites based on polyethylene with natural rubber additives have satisfactory mechanical and technological properties that determine the suitability of such materials for application as packaging and agricultural films with advanced biodegradability.

To evaluate and measure the environmental impact of the processing, exploitation and disposal of biodegradable materials, in particular PLA bottles, Baldowska-Witos et al. [9] have carefully assessed the life cycle analysis (LCA). At the initial stage, the authors collected technological data (see Table S1) covering rough resources, energy consumption, and emissions to the air, soil, and water. At the next stage, they corrected the environmental impact estimation (Table 2) including greenhouse emissions and radioactive isotopic release (Figure 4). The environmental impact of bottle production from PLA and polyethylene terephthalate was compared. In the conclusion, the authors suggest that for enhancing LCA efficiency it is necessary to carry out an analysis of key data quality and probability in relation to the results; this is presented in the next publication in this Special Issue.

The second paper of Baldowska-Witos et al. [10] is closely related to the previous publication and devoted to the in-depth development of LCA methodology. The authors propose a numerical approach to evaluate the uncertainty and precision of LCA data using stochastic modeling and sensitivity analysis. The immediate applicability of the calculation algorithm to the PLA bottle shaping procedure is based on a number of factors such as (1) the qualified and coherent evaluability of input data in matrix form, (2) the reliability of characteristics and reasonability of conclusions, and (3) the uncertainty degree of input and obtained data estimated via Monte Carlo modeling. By arranging the sequence “input data (materials and energy)—process implementation (PLA preforming, heating, cooling, etc.)—additional impact categories (fossil depletion, global warming, water consumption, etc.)”, the authors estimate the key factors that affect the eco-valuable damage category, namely human health, ecosystem quality/resistance, and resource ability.
